# A Critical Analysis of Decentralizing the Portuguese Public Healthcare Provision Services

**DOI:** 10.3390/ijerph192013390

**Published:** 2022-10-17

**Authors:** Alexandre Morais Nunes, Diogo Cunha Ferreira

**Affiliations:** 1 Centre for Public Administration and Public Policies, Institute of Social and Political Sciences, Universidade de Lisboa, Rua Almerindo Lessa, 1300-663 Lisbon, Portugal; 2 CERIS, Instituto Superior Técnico, Universidade de Lisboa, Av. Rovisco Pais 1, 1049-001 Lisbon, Portugal

**Keywords:** decentralization, national health service, regional health administration, primary healthcare, Portugal

## Abstract

The Portuguese health system has a universal, public, general National Health Service (NHS), tending towards free healthcare access. Created in 1979, this delivery model developed from the integration and complementarity between the different response levels (primary, hospital, continuing, and palliative care). However, over the last 40 years, the initially centralized system underwent a decentralization process with the creation of Regional Health Administrations in the five mainland administrative regions. Since then, the entire NHS has settled around this new organization. The most recent step started in 2018 with the decentralization of primary healthcare skills to 190 municipalities. This paper presents the various critical issues involved in the latest gradual decentralization process in health, intending to bring services closer to the citizens, and to be more focused on their needs. The article identifies and discusses the implications of this experience based on the steps foreseen in the already-published legal texts.

## 1. Introduction

Decentralization has emerged as a fundamental process of health policy-making in several European countries, especially in the Nordic countries, Switzerland, and the United Kingdom. In these countries, decentralization has become a general principle of State organization. It has also been adopted in some countries in southern Europe (France, Italy, and Portugal), although to a lesser extent [1]. The various decentralization processes in Europe played an essential structural role under the umbrella of public administration perspectives, becoming synonymous with the strengthening of regional and municipal governments in the different sectors of activity, namely in the health sector [2].

The concept of decentralization traditionally refers to a political change, shifting the central government’s responsibility to another government, regional or local, in the same country. In healthcare, decentralization occurred in several European countries, from the central government to the regional (Wales, Northern Ireland, and Scotland, for instance) or the municipal one (e.g., Finland) [2].

Nevertheless, there is no standard definition of decentralization at the conceptual (with various interpretations) or organizational (with different forms of implementation) levels. For this reason, there is not a single strategy for competencies decentralization. Instead, there is a diversification of strategies associated with many theories that are often conflicting. Rather than representing a single strategy, decentralization acts as a broad umbrella for administrative reform across countries in the most diverse sectors of activity [2]. Although there are different concepts or forms of decentralizing competencies, they share a common objective: to focus on meeting the broad needs of citizens and facilitating the public administration proximity-based processes of public policy implementation [2].

The Constitution of the Portuguese Republic of 1976 imposed an administrative regionalization that, after the 1998 referendum, became a regional and municipal functions’ reinforcement [3]. However, the decentralization processes in Portugal over the last 43 years mainly followed Rondinelli’s perspective [4,5]. They have been directed by various guidelines, including devolution and delegation or rules transfer from the central government to the regional public administration units.

In particular, the health sector in Portugal initially underwent a process of administrative decentralization at the regional level in the 1980s. This process led to the creation of five Regional Health Administrations (RHA) and their respective subregions in the various municipalities of the country. The five RHAs are North, Center, Lisbon and Tagus Valley, Alentejo, and Algarve. Although dependent on the Lisbon-based Ministry of Health, RHAs had considerable local importance and bargaining weight in the management of hospitals and health centers [6].

However, at the beginning of the 21st century, increasing signs of State inefficiency in health-service management and difficulties in access to the appropriate healthcare services led to the adoption of new public management (NPM)-related instruments. As a result, public sector accountability has increased, replacing the hierarchy with contracts or quasi-contracts in relations between the State and the public services [3,7,8]. As striking examples, the following stand out: (i) the corporatization of public hospitals, making them more autonomous from both central and regional governments, as the former became part of the State’s business sector; (ii) the introduction of public–private partnerships (PPP) for the construction, financing, and operation of public hospitals; and (iii) the contracting of services between public hospitals and private groups [6,7].

Since 2014, the national health policy and the policymakers have rethought decentralization at the primary healthcare level. The implementation of this model began in 2019, planning to deliver some responsibilities over the infrastructure, logistics, and non-technical human resources (operational assistants) of primary healthcare units to the municipalities [9]. As Nunes and Ferreira [10] point out, since 2016, the Portuguese national health policy has raised citizens’ expectations. Citizens see an improvement in providing quality care, especially in primary care and public health.

This study develops a theoretical approach to decentralization that can capture the most recent experience of transferring skills from primary healthcare to municipalities. Therefore, the paper explores several critical issues in the process faced by public health policymakers. The study proposes some strategies to improve the effectiveness of articulation among the distinct public administration structures to improve the population’s healthcare status. Indeed, the effects should have a future positive outcome on the various social and economic indicators. The article also presents a section exploring the current health decentralization process implementation. Other European countries, with predominantly public health services, may follow the contents analyzed in that section.

## 2. Materials and Methods

Decentralization applied to the health sector has sought to reduce the State’s weight in this activity area by becoming a common theme in many tax-funded health systems. This strategy began in northern Europe in the 1980s and then in western and southern Europe a decade later. The decentralization strategy has been firmly oriented towards specific areas of decision-making. Health policy-making and service organizations thus become shared exercises in which regional or local decision-makers make decisions following national guidelines. For this reason, the decentralization policy is never complete as it must balance regional or local activities and plan based on national strategies [2,11].

The 1976 Portuguese Republic’s Constitution established a system of administrative regionalization as an objective of the country’s organization [12]. However, this reform was being successively postponed by several sequential governments with different political orientations and goals. Nevertheless, some decentralization has complied with the subsidiarity model that prevailed in all EU Member States, which financed areas of activity (such as health), provided that they were implemented on a regional basis [13].

In the 1990s, the theme of regionalization (and, consequently, the associated decentralization) became the subject of national politics. It intensified the discussion around decentralization. The conclusion was that it was necessary to start the regionalization process of mainland Portugal [3]. In 1991, the Framework Law on Administrative Regions was approved [14], defining the organs of authority per region (Regional Boards and Regional Assemblies). Additionally, the law also established the competencies and rules underlying such authorities, how the regions should have been established, and the electoral regime of future areas. Only the number of areas to create and their delimitation was not defined. During the following years, there was a heated debate about the delimitation of each region. At the time of the 1997 constitutional revision, the specific establishment of areas led to a referendum. The population did not accept it in November 1998 [3].

However, this discussion period has reached an agreement on regional decentralization in the (Portuguese) health sector. The Constitution itself and the fundamental health law that proclaimed a regionalized structure for the Portuguese health system reflected this agreement. The National Health Service (NHS), incorporated into the Portuguese public health system, provides universal and general health protection for all inhabitants [15]. Through the decentralization of the NHS to the regional level, it was temporarily organized into 18 district health administrations until the definition of health regions [16]. Later, in 1993, the NHS was reorganized into five RHAs [6,17].

Reis [18] and Campos [3] considered this process an attempt to decentralize because centrally appointed regional health administrators performed the decision and management. However, the truth is that each RHA had local influence and began to operate regionally, particularly in the host city of each region. Their authority was exercised over health centers, hospitals, and other health institutions, making agreements with the private sector and adapting health plans to the resident population’s needs. It represented a significant change, as managers were appointed locally and not just by the central government [6].

A central dimension that accompanied the administrative decentralization of the RHAs was the introduction of new and innovative management tools for hospitals and other health providers [8]. Almost all of the Western European countries with tax-funded health systems, primarily Sweden (1980) and the United Kingdom (1990), had already implemented this type of NPM-based instrument [19].

Portugal and northern Italy adopted the public company model of introducing contracts for some buyer–provider divisions [2,20]. Hospitals remained public property, but with a different management model. In addition to this model of publicly owned hospitals, the Portuguese State developed PPP contracts to ensure the construction and maintenance of hospital infrastructures and universal access to healthcare [15]. In both processes, decentralized RHAs actively participated in the regulation, monitoring, contracting, and management of contracts in the case of partnerships [6].

RHAs are also responsible (1) for designing and implementing the health plan for the targeted population living in the RHA geographical area, (2) for securing agreements with the private sector, (3) for overseeing and controlling public hospitals, and (4) for managing and organizing primary healthcare, ensuring the implementation and enforcement of policies set out in the national health plan, including about public health surveillance activities [15]. However, the planning and organization of the sector of health in Portugal are the responsibility of the Health Ministry [15,21].

Table 1 presents and describes the primary sources of healthcare assistance in Portugal. Assistance can be public, private, or social, depending on provider ownership. There are four main sources of healthcare assistance—primary care, hospital care, continued care, and palliative care:

- Primary healthcare. It is the main gateway to the health system. Primary healthcare is characterized by proximity and focuses on health promotion and prevention, acute illness treatment, monitoring chronically ill patients, respecting physical, psychological, and socio-cultural dimensions, and concentrating on the patient, their family, and community [15].
- Secondary (hospital) healthcare. The level of differentiated care typically provided by public hospitals is distributed throughout the country, based on the resident population and health needs. However, hospital distribution is dependent on the existing medical professionals in certain specialties. Therefore, hospitals are classified according to the available services, providing care in terms of hospitalization, follow-up in specialty medical appointments, diagnostic and therapeutic, timely scheduled assistance in day hospital sessions, and non-scheduled emergency service [6].
- Continued integrated care. The post-hospital response level aims to provide continuity care for patients requiring effective rehabilitation with integrated support. This type of response can occur on an outpatient or inpatient basis through the severity of the health problem (convalescent care units, medium-term and rehabilitation care units, and long-term care and maintenance units) [15].
- Palliative care. Response level for end-of-life patients. It aims to support the patients and their families in a more conditioning phase that should likely lead to the end of life [15].

Both social and private health sectors in Portugal have developed from shared services with the NHS. They offer services in all specialties, namely the most profitable ones and those in which the public system is most in need [22], when the user resorts to the private sector of their own accord, they or their health subsystem or private voluntary insurance burden. As a rule, a better access issue justifies the citizens’ choice of the private sector. Other factors include comfort or inviting prices when full health insurance coverage is available. Usually, the quality of services is not a criterion [15]. However, in the case of agreements/conventions, if the NHS sends the user to a private provider, then charges are entirely borne by the State, which failed to provide the service to taxpayers [10].

The effort to foster decentralization in health occurs principally at the primary healthcare level. Indeed, this level of care is a central element of the NHS and has essential roles in health promotion and disease prevention, healthcare provision, follow-up of patients, and proximity to the population [23]. Moreover, it is the level of care closer to the citizens, distributed across all the municipalities and most parishes of the country. The RHAs have controlled this healthcare level since 1993. Since 2008, primary healthcare centers have been vertically merged or clustered into Agrupamentos de Centros de Saúde (ACES), the Portuguese words for Primary Healthcare Clusters. Each ACES is composed of family health units (USF), custom healthcare centers (UCSP), community care units (UCC), public health units (USP), and shared-resources units (URAP); see Table 2 for details [23].

## 3. Results and Discussion

The first functional relationship between primary healthcare and local authorities occurred in 1999. In that year, a law was published establishing the framework for transferring responsibilities and competencies to local authorities and the delimitation of central government and local government intervention. Thus, by implementing the principles of administrative decentralization and the autonomy of local government, this law granted new health functions to municipalities, including the participation in:

(i) the planning of the municipal health equipment network;
(ii) the construction, maintenance, and support of health centers;
(iii) the advisory bodies of establishments integrated into the NHS;
(iv) the definition of public health policies and actions carried out by the municipal health delegations;
(v) advisory bodies for monitoring and evaluation of the NHS;
(vi) the communication with citizens;
(vii) the provision of continuing healthcare within the framework of social dependency support, in partnership with the central government and other local institutions; and
(viii) to cooperate to make public health compatible with municipal development strategic planning [24,25].

In practice, this legislation was never fully implemented, with only a few municipalities supporting the health facilities in their locality, especially in election years. However, it should be mentioned that autonomy is relative. At the regional level, it is possible to carry out maintenance/construction works but with prior authorization from the central government. In other words, the region pays the expenses and is responsible for providing guarantees of good physical conditions and services, but the decision/authorization is always central to the Ministry.

More recently, the 19th Constitutional Government program [26] returned to this theme. A reforming and innovative agenda for Local Government was proposed in 2011, based on two main objectives: (i) promoting proximity with citizens and (ii) administrative decentralization. This strategy intended to replace the centralist and macrocephalus paradigms with a responsibility paradigm. It also planned to value the efficiency in allocating resources to the social, economic, cultural, and environmental development of the various regions of Portugal. This paradigm was based on the principles of subsidiarity in deepening municipalism, strengthening the competencies of municipalities’ associations, and promoting territorial cohesion and competitiveness [26].

To this end, the 19th Constitutional Government aimed to develop competency, financing, and resource transfer models and new perspectives on local organizations to endorse the transfer of competencies and resources from central government to municipalities. Regarding health, the government’s priority focused on strengthening the municipalities’ participation in planning the national network of health equipment [26].

Despite the intention, this transfer of competencies did not actually occur. Five years later, the issue of decentralization and administrative reform was also on the agenda of the 20th Constitutional Government Program [27]. It undertook to continue the process of decentralizing competencies, ensuring (i) efficiency gains and (ii) the transfer of human resources, material, and financial resources necessary for the exercise of decentralized controls.

However, the 21st Constitutional Government established the implementation of decentralization as the cornerstone of state reform, as embodied in the Constitution of the Portuguese Republic. In this context, the government sought to strengthen the competencies of local authorities [28], based on the best interests of citizens and the promotion of equity in access to appropriate healthcare and disease prevention.

The 21st government implemented such a transfer of competencies. In 2018, the Framework Law on the transfer of competencies to local and inter-municipal entities was published [29]. It was based on the principles of subsidiarity, administered decentralization, and local government autonomy. The framework for the competencies transfer to municipal organs and the specific inter-municipal entities in the health domain was completed in 2019 [9].

The main goal of health skills transfer was to improve the public service through (i) development of excellence-based projects, (ii) innovation, and (iii) more effective and measurable responses that enable increased community involvement in the management of primary healthcare and strengthening the accountability of different entities for the quality of the health service [9].

Competencies transfer is key to an articulated and integrated management model of primary healthcare in the municipal territory through:

(i) the promotion of both effectiveness and efficiency of health resource management in achieving better health outcomes within the municipality;
(ii) the creation of synergies from local community involvement in healthcare delivery; and
(iii) the articulation between the various levels of Public Administration [9].

Given the government’s program [28] and legislation [9,29], the competencies transferred to municipal bodies include:

(i) the participation in the planning, management, and investment of new primary healthcare units, including their construction, equipment, and maintenance;
(ii) the management, maintenance, and conservation of already-existing primary healthcare equipment;
(iii) the management of operational assistants that currently belong to the staff of each functional unit from each ACES (see Table 2);
(iv) the services related to logistics support for the ACES functional units; and
(v) the strategic partnership in health programs (supporting disease prevention, healthy lifestyles, and active aging).

Given the generality of the transferred competencies, it was necessary to specify each one of these areas in more detail. Table 3 details the competencies assigned to municipalities in the health decentralization process for primary healthcare, in terms of participation in investment planning, management, and realization, logistics management of ACES functional units’ support services, management of operational assistants, and strategic partnership in health programs. From an analysis of this table, one may conclude that, virtually, the municipality bears all costs, except those related to health professionals and logistic support services associated with medical equipment. These remain within the sphere of the central government as part of NHS funding. In contrast, municipalities acquire ownership of facilities and equipment, except medical equipment.

This transfer of ownership obliges municipalities to ensure the quality of interventions and the optimal operation and safety conditions. In contrast, the Ministry of Health must verify these same conditions, safeguarding the interest of both users and health professionals.

Its scope highlights this process of competencies transfer. It comprises 190 municipalities, 46 ACES, and 8,884,071 citizens from the five RHAs within the national territory (see Table 4).

The legislation is unclear regarding the payment of expenses associated with the management and implementation of logistics support services. Similarly, the law also does not appear clear about the costs related to the new employer’s (the municipality’s) human resources burden. Concerning maintenance expenses, the diploma mentioned above highlights the annual transfer to the cities of a sum to be included in the Decentralization Financing Fund to pay facilities’ maintenance costs, resulting from the following formula: value per square meter × gross building area [9].

Regarding the monitoring and follow-up of the process, the legal document [9] is more transparent in highlighting the creation of a commission per municipality. This committee consists of the mayor, a representative of the corresponding RHA, and a representative of the related ACES. This committee should monitor the implementation of health competencies decentralization and propose adopting additional measures if deemed necessary to pursue the proposed objectives.

In short, the decentralization process in Portugal in healthcare, after the whole process, only transferred the infrastructure maintenance (construction/repair), service provision (transport, electricity, water, sanitation, security, non-clinical equipment, and support staff (not health professionals)). Therefore, in terms of funding for the provision of healthcare, nothing has changed. Hospitals were not included in the process. Portugal did not risk a total or complete transfer. Everything concerning the clinical area remains centralized in the Ministry of Health. In terms of structures, a larger municipality needs more structures, but it also has greater economic power. However, being an investment mainly for the structure, it ends up being proportional to the size of the municipality.

## 4. Conclusions

Significant steps toward achieving the right for all to access healthcare in Portugal began during the 1970s because of the publication of the Portuguese Republic Constitution and the introduction of a centralized NHS.

In its first 40 years of existence, the NHS was managed by a national health policy centrally directed by the Ministry of Health and by a set of regional/local healthcare providers working under the direction of the RHAs and with a group of private providers. Although there have been some improvements in the population’s health status, it is still necessary to reinforce proximity to the citizen [7,8,10].

According to the literature [31], decentralization in the health system has advantages and disadvantages. The democratic argument assumes that decision-making should be closer to people, meet their health-related needs, and provide better health responses [32]. In opposition, regional asymmetries in countries where the Constitution defends the right to health for all citizens are a potential disadvantage of the model [6].

Decentralization in Portugal has been present since the 1976 Constitution. The creation of RHAs and the transfer of management competencies to municipalities, which deal directly with citizens, can be considered two critical milestones. Health decentralization in the NHS with the creation of RHAs was never intended to be followed by a privatization movement. Instead, it was a public administration tool influenced by the NPM’s perspective, reflecting a response to external pressure to improve the quality and efficiency of the NHS providers.

The first stage of decentralization was never a movement toward privatization or pure regionalization (as in Spain, for example), and was only seen as a tool of public administration. Nonetheless, Bafoil and Hibou [13] criticized it, dubbing it a “false decentralization” because (i) it was created to cover financial circuits to manage EU programs, (ii) it had a weak regional organization due to a structure centrally influenced by the Ministry of Health, which maintained its influence (iii) on manager appointments, (iv) in the allocation of financial resources, (v) in the allocation of human resources, (vi) in financing decisions, and (vii) in the organizational rules.

In the second significant decentralization stage, and from a proximity perspective, joint action should improve the response to citizens (at the primary healthcare level). This action focuses on the construction, management, and maintenance of infrastructures, logistics issues, the management of non-clinical human resources, and the health strategy in terms of disease prevention and health promotion.

The model was started in late 2019 and suspended in 2020 due to COVID-19. In this short time, there was no opportunity to evaluate it. One should remember that the transfer of services and the involvement of regions and municipalities are necessary in the decision-making process. Thus, we suggest that the decentralization process should be monitored and reviewed. It should be in line with Harding and Preker [33], who consider the focus of decentralization to be on health, not merely the transfer of services. Instead, the focus should be on decision-making control and, often, on the rights and responsibilities of central government agencies for lower levels.

## Figures and Tables

**Table 1:** Sources of healthcare assistance in Portugal [10].

Assistance	Public	Private	Social
Primary healthcare	Healthcare centers (including public health activities) Local Health Units	Clinics (general medicine and occupational medicine appointments)	Offices (general medicine and occupational medicine appointments)
Secondary healthcare	Hospitals Hospital centers Local Health Units	Hospitals Clinics	Hospitals Clinics
Continued healthcare	Convalescence Units Medium Duration and Rehabilitation Units Long-Term and Maintenance Units Home Support Teams		
Palliative care	Continuing Care Units Support Teams		

**Table 2:** Characterization of the functional units belonging to the Portuguese primary healthcare groups.

Functional Unit	Characteristics
Family health unit (USF, standing for the Portuguese words Unidade de Saúde Familiar)	Healthcare unit devoted to both individual and family care and based on multidisciplinary teams of physicians, nurses, and administrative staff. There are three USF models, differentiating themselves in terms of organizational autonomy degree, remuneration model, incentives to the staff, financing model, and legal status.
Custom healthcare center (UCSP, standing for the Portuguese words Unidade de cuidados de saúde personalizados)	The structures of UCSP and USF are similar. UCSPs provide personalized care, ensuring full access to all citizens as well as the continuity and comprehensiveness of healthcare services. The UCSP team is composed of non-USF physicians, nurses, and administrators.
Community care unit (UCC, standing for the Portuguese words Unidade de cuidados na comunidade)	Healthcare unit providing healthcare and psychological/social support, at home and in the community, especially to the most vulnerable people, at higher risk, either dependent or with a disease requiring close monitoring. The UCC team consists of nurses, social workers, doctors, psychologists, nutritionists, physiotherapists, speech therapists, and other professionals. Through the UCC, the ACES participates in the National Network of Integrated Continuing Care, integrating the local coordinating team.
Public health unit (USP, standing for the Portuguese words Unidade de saúde pública)	The USP is a unit working as a health observatory for the ACES of which it is part. In particular, it is responsible for preparing public health information and plans, conducting epidemiological surveillance, and managing prevention, as well as promotion and protection intervention programs. The USP team is composed of public health doctors, public health/community health nurses, and environmental health technicians, as well as other professionals deemed necessary in the public health area.
Shared resources unit (URAP, standing for the Portuguese words Unidade de recursos assistenciais partilhados)	The URAP provides consulting and assistance services to the previous functional units, and organizes functional links to hospital services. The URAP team is composed of doctors from various specialties other than general/family medicine and public health, as well as other staff, including social workers, psychologists, nutritionists, physiotherapists, and oral-health technicians.

**Table 3:** Competencies assigned to municipalities in the health decentralization process for primary healthcare [9,29].

(i) Participation in investment planning, management, and realization	To invest in new primary healthcare units, namely in their construction and equipment, always preceded by a binding prior opinion from the Government stakeholder responsible for health and healthcare programs
	To promote programs of financial support for investment operations in primary healthcare units, either through State Budget appropriations or through the allocation of capital from European Structural and Investment Funds
	Management, maintenance, and conservation of primary healthcare facilities and equipment; risk-sharing in additive behaviors’ intervention; reduction of dependence on regional health administrations
	To ensure the quality of the healthcare provided, as well as the proper operating and safety conditions of the facilities
	To provide the Ministry of Health with the information necessary to carry out its duties, so that it can monitor the execution of the services provided and verify that the necessary and appropriate conditions for healthcare activities are being observed
(ii) Logistics management of ACES functional units’ support services	Cleaning services
	Surveillance and security support activities
	Electricity, gas, water, and sanitation supply
	Vehicles and related insurance, fuel, compulsory periodic inspection, and maintenance charges
	Travel expenses, when used for healthcare
	Health insurance
	Lifts maintenance and conservation
	Maintenance of heating, ventilation, and air-conditioning systems
	Payment of rent and other charges, when applicable
(iii) Management of operational assistants	The transition of publicly employed workers from the staff of the Regional Health Administrations to the staff of each municipality
(iv) Strategic partnership in health programs	To develop or participate in disease-prevention/health-promotion activities (healthy eating, regular exercise, and active aging), in partnership with the regional health administration, under the corresponding action plan as well as each municipal health plan
	To link home-based social activities with health interventions within the primary healthcare units and the National Integrated Continuing Care Network
	To promote the health of women, children, and adolescents, as well as diabetes prevention
	To implement mobile health-intervention units

**Table 4:** Entities participating in the health decentralization process [29,30,31].

	Councils	Health Center Clusters	Citizens
North	61	21	3,125,804
Center	53	6	1,583,093
Lisbon and Tagus Valley	49	15	3,557,442
Alentejo	13	1	166,726
Algarve	14	3	451,006

## Data Availability

Not applicable.
